# Corrigendum to “Early Detection of Medical Image Analysis by Using Machine Learning Method”

**DOI:** 10.1155/2022/9878749

**Published:** 2022-07-05

**Authors:** Ahmad M. Khasawneh, Amal Bukhari, Mahmoud Ahmad Al-Khasawneh

**Affiliations:** ^1^Department of Cybersecurity, Amman Arab University, Amman 11953, Jordan; ^2^College of Computer Science and Engineering, University of Jeddah, Saudi Arabia; ^3^School of Information Technology, Skyline University College, University City Sharjah, 1797 Sharjah, UAE

In the article titled “Early Detection of Medical Image Analysis by Using Machine Learning Method” [1], there was an error in the caption for Figure 1. The authors would like to clarify that Figure 1 was reproduced from Chaddad et al. (2021) [2].
